# Transverse process fractures of the thoracic vertebrae—the significance of this injury in the context of medicolegal opinions on high-energy trauma cases

**DOI:** 10.1007/s00414-019-02161-7

**Published:** 2019-09-16

**Authors:** Aleksandra Borowska-Solonynko, Victoria Prokopowicz

**Affiliations:** Department of Forensic Medicine, Medical University of Warsaw, Oczki 1, 02-007 Warszawa, Poland

**Keywords:** Thoracic transverse process fractures, Postmortem computed tomography, High-energy trauma, Traditional autopsy

## Abstract

Thoracic transverse process fractures (TTPFs) are injuries that go unnoticed during traditional autopsies, as demonstrated by a lack of medicolegal publications regarding TTPFs. However, postmortem computed tomography (PMCT) has made detection of this type of injury easy. Thus, the goal of our study was to analyze the significance of TTPFs in the context of medicolegal opinions. Forensic autopsy reports and PMCT scans of 116 people who had died from high-energy trauma were analyzed. TTPFs were found in 34.48% (*n* = 40) of the total test group. The highest proportions of TTPFs were found in drivers (50%, *n* = 8) and in victims of falls from heights (41%, *n* = 14). Among seven car passengers, only one victim had suffered TTPFs. In comparison with persons without TTPFs, persons with TTPFs demonstrated more severe general injuries, especially to the chest and abdomen, and more often (in 90% of cases) died at the scene of injury (all these differences were statistically significant; *p* < 0.0001). Pedestrian TTPFs were present only in victims struck from their front or back. TTPFs in victims of falls were found only in those cases in which the height of the fall was at least 9 m. The presence of TTPFs indicates that the application of a very strong force leads to injuries that, in most cases, result in death at the scene of the event. Detecting TTPFs provides additional information about the mechanism of trauma, especially in pedestrians, drivers, passengers, and victims of falls from heights.

## Introduction

In medicolegal practice, determining the cause of death in victims of high-energy trauma is usually not difficult. Problems, however, may appear during reconstruction attempts. In these situations, a detailed description of the injuries and knowledge of the mechanisms that lead to them are essential. Thanks to the more and more widespread use of postmortem computed tomography (PMCT), it has become possible to diagnose injuries (especially bone injuries) which, until now, had been very easy to overlook during traditional autopsies [1–6]. One type of such injuries, which are very difficult—and in many cases, impossible—to detect during a traditional autopsy, are thoracic transverse process fractures (TTPFs). During a traditional autopsy, ventral access to thoracic transverse processes is hindered by the ribs and dorsal access by massive muscles of the back. Therefore, according to our knowledge, there are no medicolegal publications dealing with this injury. *The purpose of our work was to determine whether detecting TTPFs can be helpful in determining the circumstances and mechanism of injuries.* The starting points for this study were clinical studies dealing with the problem of transverse process fractures (TPFs) and emphasizing the importance of this finding in the context of comorbidity with other serious injuries [7–10]. In most of these publications, the authors stress that TPFs are a result of high-energy trauma [7]—hence, we chose these types of cases for our analysis.

## Material and methods

### The study group

**Fig. 1:**
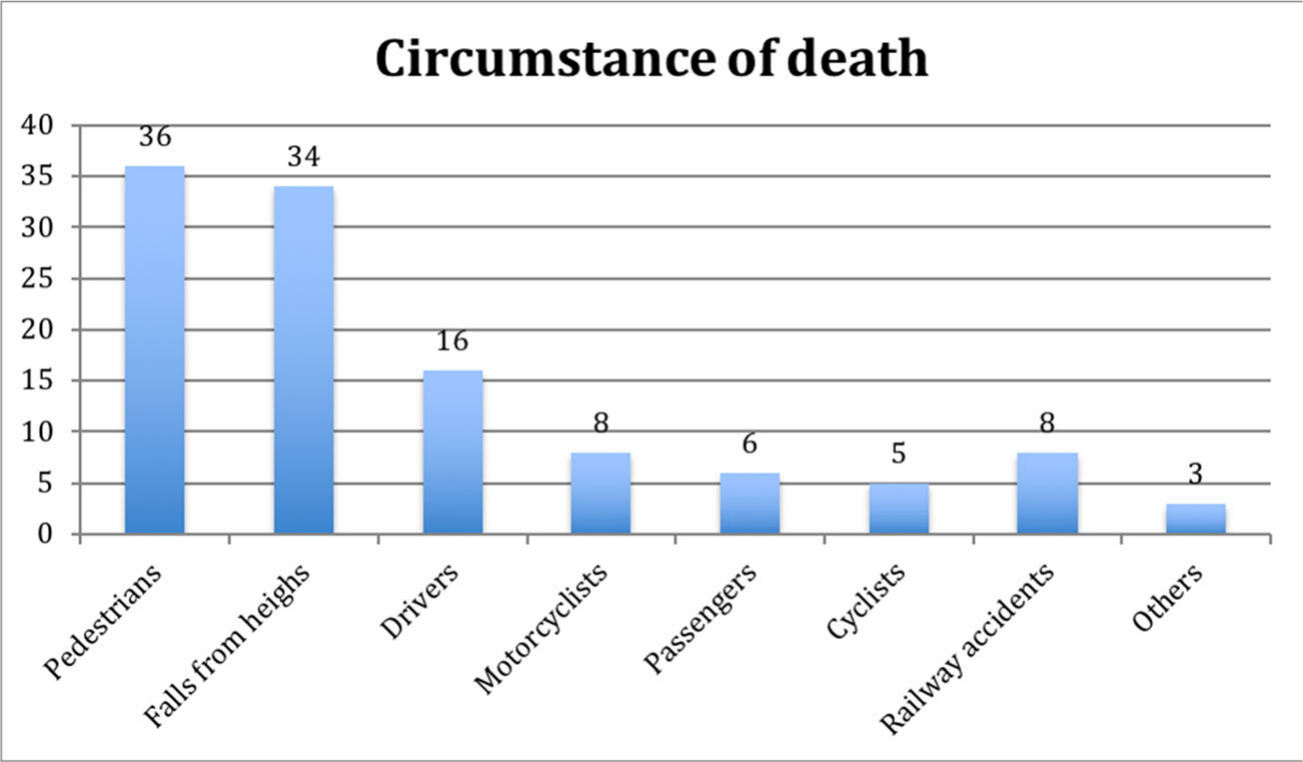
Circumstances of death (numbers of cases)

This retrospective case-control observational study was conducted based on our data regarding the persons who had died as a result of high-energy trauma (i.e., in circumstances indicating that the severity of injuries was due to a high-energy impact) and whose forensic autopsies were performed in the years 2014–2016. The study included only those cases in which PMCT was performed and a postmortem report was available. Initially, 141 decedents were included in our study. After excluding those cases where complete autopsy data was not available and those cases where the decedent was under 18 years old, the total study sample comprised 116 people aged 18–93 years (mean age 49.76 years). This group consisted predominantly of men, who made up 74.13% (*n* = 86). Considering the circumstances of death, the largest groups were pedestrians (31.03%, *n* = 36) and victims of falls from heights (29.31%, *n* = 34). The numbers and proportions referring to other circumstances of death are presented in Fig. 1.

### PMCT acquisition and evaluation

PMCT scans were obtained with a 16-row Astelion CT scanner (Toshiba). In each case, unenhanced CT scans were performed, with 1-mm-thick slices acquired at 120 V with automatic exposure control (AEC). The pitch factor was 1.438 for the trunk and 0.688 for the head. Scan field-of-view (FOV) for the head was maximum 320 mm; for the trunk and lower limbs, it was between 390 and 500 mm. Cadavers were scanned in a supine position with the standard protocol, including acquiring scans of the head with neck, torso, and lower limbs (when needed). The examination was performed without opening the protective plastic bag or altering the position of the corpse inside. Three different reconstruction kernels were used for image acquisition (Toshiba FC30 for bones, FC18 for soft tissue, and FC26 for the brain). The PMCT scans were analyzed using Osirix application (Osirix MD v.0.8.1) with the use of bone and soft-tissue window settings, whereas TTPFs were assessed only in bone window settings. The assessment was conducted independently from autopsy findings. Each CT scan was assessed by a board-certified forensic pathologist with experience in forensic radiology, and all ambiguities were consulted with a board-certified radiologist.

### Analyzed data

**Fig. 2:**
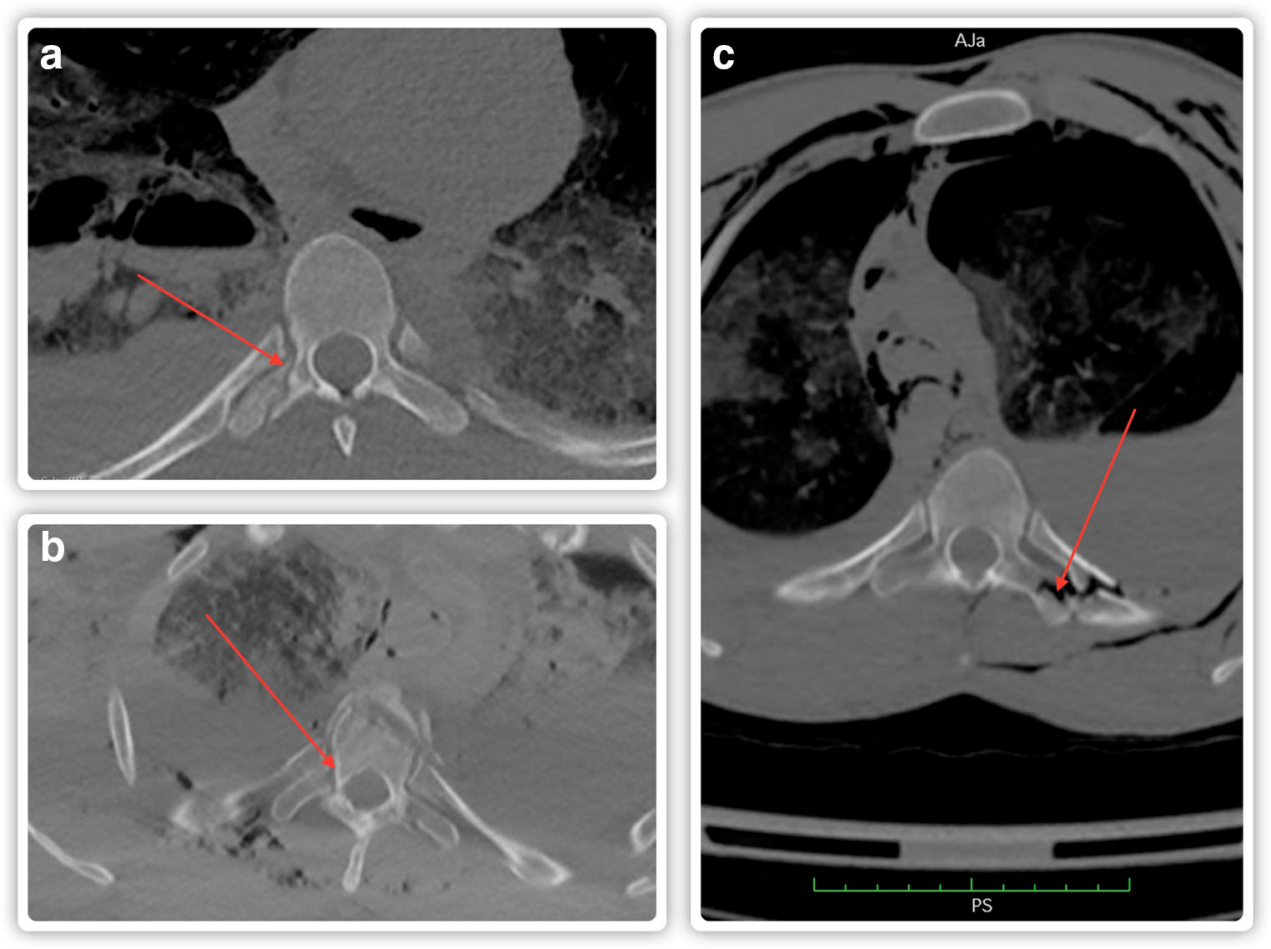
Postmortem computed tomography (PMCT) multiplanar reconstruction (MPR) images (axial slices) presenting thoracic transverse process fractures (TTPFs)—marked with arrows. **a** Isolated fracture. **b** Complex fracture. **c** Fracture accompanied by rib fractures

The following data were analyzed: age; sex; mechanism and circumstances of death; cause of death; blood ethanol concentration at the time of death, whether or not the person was hospitalized; number of days the person survived after sustaining trauma; whether or not there were injuries to the chest and abdomen or pelvis; vertebral fractures in the cervical, thoracic, and lumbar spine; and the presence or lack of TTPFs. Bone injuries were defined as any fractures or cases of joint separation. Internal organ injuries were defined as evidence of any trauma (e.g., ruptures, extravasations) recorded in the autopsy report, irrespective of their extent or severity. The observed TTPFs were divided into the following: isolated, complex (i.e., part of a vertebral fracture), accompanying rib fractures (Fig. 2), and mixed (more than one type in one person). In the TTPF group, we determined the side (bilateral, right, left) and a possible lateralization of the fractures (in the cases where fractures were bilateral, the side with more fractures was indicated), as well as the level of the thoracic spine affected (upper T1–T6, lower T7–T12, middle whenever fractures were present in consecutive vertebrae in the midsection of the thoracic spine, i.e., T5–T8, or multi-part whenever fractures were present in various sections of the thoracic spine) and the number of transverse process fractures (one, two, multiple). In addition, the severity of injuries was assessed in each case using the Abbreviated Injury Scale (AIS) for the head and neck, chest, abdomen, and extremities; the Injury Severity Score (ISS); and New Injury Severity Score (NISS). The NISS is calculated as the sum of the squares of the top three scores, regardless of the body region. The above data was acquired from PMCT scans, autopsy reports, and the descriptions contained in prosecutorial orders for performing an autopsy. The data on TTPFs contained in PMCT reports and autopsy reports was impossible to compare, as none of the forensic pathologists performing the traditional autopsy (and composing the relevant report) had mentioned any TTPFs. Although mineral bone density was not measured in any of the cases, none of the reviewed autopsy reports mentioned excessive bone fragility.

### Statistical analysis

TIBCO Software Inc. (2017) Statistica (data analysis software system), version 13, was used for statistical analysis. The chi-square test and Fischer exact test were applied to compare groups of categorical variables. The influence of continuous data on the study groups was assessed using a two-sample *t* test for parametric variables or the Mann-Whitney U test and the Kruskal-Wallis H test (i.e., one-way ANOVA on ranks) for non-parametric variables. The chi-square or Kolmogorov-Smirnov tests were used in order to determine the distribution of data. The results were considered statistically significant when the adjusted *p* values were less than 0.05 (*p* < 0.05). The following correlation coefficients were also used: coefficient *Φ* and the contingency coefficient *C*. Log-linear analysis was used to test the mutual influence of various qualitative factors.

## Results

### Total study group calculations

**Fig. 3:**
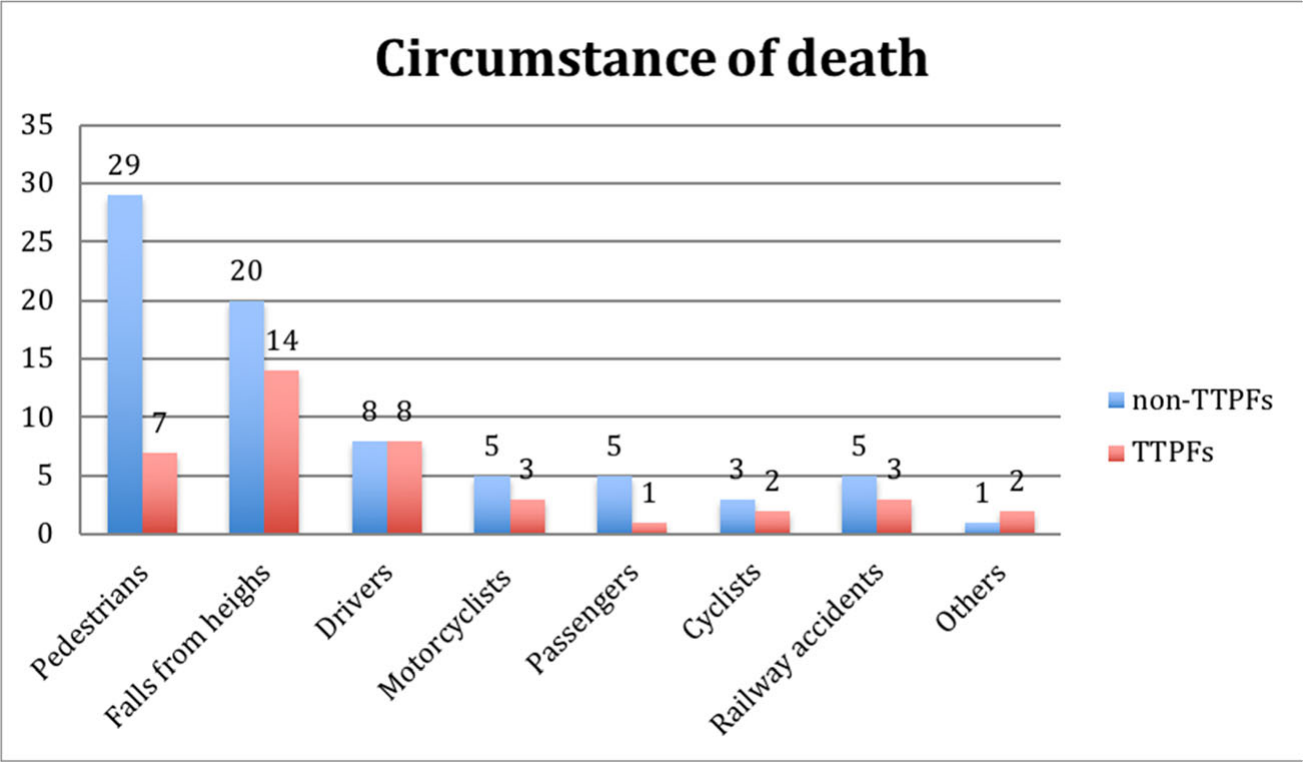
Circumstances of death stratified by the presence or absence of thoracic transverse process fractures (TTPFs)

*TTPFs were found in 34.48%* (*n* = 40) of the whole study group. Apart from a very small number of deaths, whose circumstances were identified as “other” (two cases of crushing injury at work and one due to being crushed and pinned between vehicles), the highest percentage of TTPF cases was found in drivers (50%, *n* = 8) and in victims of falls from a height (41.17%, *n* = 14) (Fig. 3). *Of the six passenger cases, only one person had a TTPF.* The relationship between the circumstances of death and the presence of TTPFs is not statistically significant, but this may be a result of the very low numbers of cases in each subgroup. In 92.5% (*n* = 37) of cases with TTPFs, multi-organ injuries were reported as the cause of death.

There was no statistically significant relationship between the group with TTPFs and without this type of fracture (non-TTPFs) in relation to the victim’s sex (men constituted 75% of the TTPF group and 73.68% of the non-TTPF group), age, blood ethanol concentration, or head and limb injury evaluated with the AIS scale. In contrast to non-TTPF cadavers, those with TTPFs had considerably more severe injuries to the chest and abdomen (rated with the AIS scale) and more severe general injuries assessed with the ISS and NISS scales (Table 1). These differences were statistically significant. Persons from the TTPF group also survived for a significantly shorter period of time following the incident than those from the non-TTPF group (Table 1). *The majority of persons with TTPFs died at the site of the incident* (90%, *n* = 36), as opposed to those without TTPFs, with 43.42% (*n* = 33) of the latter subgroup dying at a later time in a hospital.

**Table 1:** Comparison of age, blood alcohol concentration, severity of injuries, and time from trauma to death in relation to the presence or absence of thoracic transverse process fractures (TTPFs vs. non-TTPFs)

	TTPFs			Non-TTPFs			*p* value
	Mean	Min	Max	Mean	Min	Max	
Age (years)	45.48	18	83	51.77	19	93	*p* > 0.05
Alcohol ‰	0.37	0	2.7	0.68	0	5.5	*p* > 0.05
AIS head and neck score	3.7	0	6	3.38	0	6	*p* > 0.05
AIS chest score	*5.12*	*2*	*6*	*3.57*	*0*	*6*	*p < 0.0001*
AIS abdomen score	*3.7*	*0*	*6*	*2.05*	*0*	*5*	*p < 0.0001*
AIS extremity score	2.2	0	6	1.62	0	5	*p* > 0.05
ISS	*59.66*	*20*	*75*	*45.80*	*9*	*75*	*p < 0.0001*
NISS	*65*	*20*	*75*	*49.9*	*9*	*75*	*p < 0.0001*
Time from trauma to death (days)	*0.1*	*0*	*3*	*1.89*	*0*	*20*	*p = 0.01*

Statistically significant results are in italics. *AIS*, abbreviated injury scale; *NISS*, new injury severity score; *ISS*, injury severity score

Table 2 presents a list of selected injuries to the chest and abdomen, pelvis, and spine, depending on the presence or absence of TTPFs. There was a statistically significant relationship between the presence of TTPFs and rib fractures. All TTPF cases were accompanied by rib fractures. Lung damage was also present in 95% of the TTPF cases versus only 63.16% (*n* = 58) of the non-TTPF group. A more pronounced relationship was found between the presence of TTPFs and heart injuries—70% (*n* = 28) of TTPFs were accompanied by an injury to the heart, in contrast to only 16.42% (*n* = 14) of non-TTPFs. Moreover, this relationship showed a high correlation coefficient (*Φ* = 0.51). There was also a statistically significant relationship between the presence of TTPFs and injuries to the thoracic aorta. Here, the correlation coefficient was clearly lower than in the case of heart injuries (*Φ* = 0.34). A combined analysis of TTPFs, heart injuries, injuries to the thoracic aorta, and thoracic spine fractures indicated two strong relationships—the first one between TTPFs and heart injuries and the other one between thoracic aorta injuries and fractures of the thoracic spine. A separate analysis confirmed that there was a strong, statistically significant correlation between injuries to the thoracic aorta and spinal fractures in this area (*p* < 0.0001, *Φ* = 0.43). Another statistically significant relationship was found between TTPFs and injuries to the liver and to the spleen, with spleen injuries yielding a higher correlation coefficient (*Φ* = 0.34) than liver injuries (*Φ* = 0.22). Intestinal and mesenteric injuries were found to have a statistically significant relationship with TTPF versus non-TTPF cases. There was also a significant relationship between abdominal aortic injuries and TTPFs, with all three cases of damage to the abdominal aorta accompanied by TTPFs. A combined analysis of the co-occurrence of TTPFs and selected injuries to the abdomen (log-line analysis), including injuries of the liver, spleen, kidneys, and intestines, indicated a strong relationship only between TTPFs and spleen injuries.

**Table 2:** Comparison of the presence of selected organ injuries, injuries of the spine, and pelvic fractures in relation to the presence or absence of TTPFs

Internal injuries	TTPFs	Non-TTPFs	*p* value
Rib fractures	*100% (n = 40)*	*81.58% (n = 62)*	*p = 0.0038*
Lung injuries	*95% (n = 38)*	*63.16% (N = 48)*	*p = 0.0002*
Heart injuries	*70% (n = 28)*	*16.42% (n = 14)*	*p = 0.0001*
Thoracic aortic injuries	*55% (n = 22)*	*21.05% (n = 16)*	*p = 0.0002*
Liver injuries	*68% (n = 27)*	*43.42% (n = 33)*	*p = 0.0136*
Spleen injuries	*60% (n = 24)*	*25% (n = 19)*	*p = 0.0002*
Kidney injuries	30% (*n* = 12)	16% (*n* = 12)	*p* > 0.05
Abdominal aortic injuries	*7.69% (n = 3)*	*0*	*p = 0.0143*
Intestinal injuries	*38% (n = 15)*	*19.74% (n = 15)*	*p = 0.0379*
Cervical spine injuries	28% (*n* = 11)	21.05% (*n* = 16)	*p* > 0.05
Thoracic spine injuries	*63% (n = 25)*	*19.74% (n = 15)*	*p < 0.0001*
Lumbar spine injuries	*25% (n = 10)*	*9.21% (n = 7)*	*p = 0.0223*
Pelvic fractures	48% (*n* = 19)	46.05% (*n* = 35)	*p* > 0.05

Statistically significant results are in italics

Our analysis of fractures in the cervical, thoracic, and lumbar spine and in the pelvis revealed a higher rate of statistically significant relationships between injuries of the thoracic and lumbar spine in the TTPF group compared with that in the non-TTPF group. There was no such relationship found in relation to cervical spine or pelvic fractures.

Based on autopsy reports, it was also determined that injuries indicating direct trauma to the dorsal aspect of the body were statistically more significant in the TTPF group (94%; *n* = 34) than in the non-TTPF group (65%; *n* = 49). There were only two cases in the TTPFs group in which no evidence of back injury was found: one cyclist who was hit by a truck and one car passenger.

### Analysis of the TTPF group

Separate analyses were carried out for the subgroup with identified TTPFs. Due to the large total number of analyses, below we present only those results that were statistically significant or interesting (though not statistically significant).

*In most cases (52.5%; n = 21), TTPFs were present bilaterally*. In 25% of cases (*n* = 10), TTPFs were found unilaterally on the right side and in 22.5% of cases (*n* = 9) unilaterally on the left side, which shows no predilection for one specific side of the body. Multiple fractured transverse processes were the most common occurrence (75%, *n* = 30). In 15% of cases (*n* = 6), two processes were fractured. The remaining four cases demonstrated a single transverse process fracture. Three out of the four persons with a single TTPF were car drivers, while the fourth person died after being hit and crushed by an excavator bucket at a construction site. Mixed type fractures (40%, *n* = 16) or TTPFs with a direct correlation to rib fractures (25%, *n* = 10) were found in the majority of cases. There were seven isolated and complex fractures, and they accounted for 17.5% of TTPF fractures. More detailed characteristics of persons with isolated TTPFs are presented in Table 3. This subgroup was dominated by young persons and victims of falls from heights. Apart from a solitary case of fracture at a single level of the spine, most likely due to a direct impact to that area by an excavator bucket, the remaining TTPFs were multilevel and most of them were found in the lower vertebrae. A tendency towards right-sided lateralization of isolated TTPFs was also confirmed.

**Table 3:** Characteristics of persons with isolated TTPFs

No.	Mechanism of death	Circumstances	Age	TTPF level	Side of the fracture	Additional autopsy findings and other details
1.	Driver	Car-train collision	83	T6–T9	Right	Pathologic spine mobility at the level of T6–T8, back injuries
2.	Fall from height	Fall from a BTS (base transceiver station) tower	18	T6–T10	Bilateral	Back injuries
3.	Fall from height	Fall from a bridge onto concrete	23	T9–T12	Right	Back injuries
4.	Fall from height	–	–	T3–T9	Right	Occipital condyle fractures, back injuries
5.	Death at a construction site	Hit and crushed by an excavator bucket	29	T12	Right	Severe abdominal injuries, L2 vertebral fracture, back injuries
6.	Passenger	The car hit a tree	22	T1–T9	Left	The passenger had clear lateralization of injuries to the left, no back injuries. He died at the scene. (The driver died in a hospital)
7.	Fall from height	8th floor (7 levels above the ground story)	78	T1–T5	Right	Back injuries

Our analysis of the sections of the thoracic spine affected by TTPFs showed predominantly *multifocal fractures (55%, n = 23) and fractures in the upper thoracic segment (20%, n = 8).*

The statistically significant relationships were found between the side of TTPFs and the circumstances of death (*p* = 0.043) and between the side of TTPFs and lateralization of injuries (*p* = 0.007). However, bilateral TTPFs were usually found in victims of falls from heights, pedestrians, motorcyclists, and victims of railway accidents, while one-sided fractures dominated in drivers, with 50% of them (*n* = 4) being right-sided and 37.5% (*n* = 3) left-sided. Cases of bilateral TTPFs were usually accompanied by a lack of lateralization of other injuries.

There was a statistically significant correlation between the presence of thoracic aortic injuries and the type of TTPF (*p* = 0.037), with 86% of aortic injuries accompanied by complex fractures, and only one case of aortic injury accompanied by an isolated TTPF.

Table 4 presents the relationship between the section of thoracic spine affected by TTPFs and the circumstances of death; this relationship was statistically significant (*p* = 0.028, *Φ* = 0.68). Most circumstances of death were produced by multifocal TTPFs. with upper thoracic segment fractures predominant only in drivers who died in traffic accidents.

**Table 4:** TTPF location stratified by the circumstances of death

	Multifocal TTPFs	Upper segment TTPFs	Lower segment TTPFs	Middle segment TTPFs
Falls from height	79% (*n* = 11)	14% (*n* = 2)	7% (*n* = 1)	0
Traffic accidents (drivers)	25% (*n* = 2)	50% (*n* = 4)	25% (*n* = 2)	0
Traffic accidents (passengers)	100% (*n* = 1)	0	0	0
Traffic accidents (pedestrians)	57% (*n* = 4)	29% (*n* = 2)	0	14% (*n* = 1)
Motorcycle accident	33% (*n* = 1)	0	0	67% (*n* = 2)
Cycling accident	50% (*n* = 1)	0	50% (*n* = 1)	0
Railway accidents	100% (*n* = 3)	0	0	0
Other	0	0	50% (*n* = 1)	50% (*n* = 1)

Due to the potential for using the diagnosis of TTPFs for incident reconstruction purposes, separate additional analyses were performed for selected circumstances-of-death subgroups: victims of traffic accidents (pedestrians, drivers, and passengers) and victims of falls from heights. The results are presented below. Due to the small number of cases, no individual statistical analysis was performed for any subgroup.

### Analysis of the pedestrian subgroup

*TTPFs were found in only 19% of pedestrians.* On the basis of autopsy findings, with particular attention paid to the description of injuries to the lower limb, the direction of impact could be estimated in 93.01% (*n* = 27) of non-TTPF pedestrians and in 71.42% (*n* = 5) of pedestrians with TTPFs. All persons in the TTPF group were found to have been hit either from their front (*n* = 2) or back (*n* = 3); this was in contrast to the non-TTPF pedestrian group, where sideways impacts dominated (*n* = 22). In the non-TTPF group, there was no one who had been hit from their front. There were, however, three persons who had been hit from their back, one of whom had died in the same accident as another pedestrian (who did have TTPFs) after a car had plowed into people standing at a bus stop. The difference between these two pedestrians was such that the pedestrian with TTPFs was hit from the front and sustained injuries to the heart, while the pedestrian without TTPFs was hit from the back and did not sustain heart injuries. *The TTPF group did not include any cases of people who had been run over.* Pedestrians with TTPFs were more seriously injured, with the most apparent difference being the severity of abdominal injuries (Table 5). All pedestrians with TTPFs died at the scene of the incident, while 51.72% (*n* = 15) of those from the non-TTPF group survived long enough to die in a hospital.

**Table 5:** Severity of injuries in pedestrians, drivers, and victims of falls from height in relation to the presence or absence of TTPFs

	Pedestrians		Drivers		Falls from heights	
Severity of injuries	TTPFs	Non-TTPFs	TTPFs	Non-TTPFs	TTPFs	Non-TTPFs
AIS head and neck score	4.14	3.41	3.87	3.12	3.21	3.10
AIS chest score	5.14	3.34	5.37	3.62	5.0	3.78
AIS abdomen score	4.28	1.75	3.75	2.25	3.21	2.63
ISS	64.57	43.93	67.62	50.75	52.92	45.26
NISS	65.85	46.20	68.75	56	62.35	49.63

*AIS*, abbreviated injury scale; *NISS*, new injury severity score; *ISS*, injury severity score

### Analysis of the drivers and passengers

*As many as 50% (n = 8) of the drivers had TTPFs, with only one out of the six passengers having suffered this type of injury* (a short description of this case can be found in Table 3—person number 6). Unfortunately, there was no data to help determine whether or not that person had had their seatbelts fastened. Among the drivers with TTPFs, in the case of four, the car each had been driving collided with a truck; in one case, the car collided with a streetcar; and in another case, the car hit a tree, was deflected back onto the road (with the driver inside), and collided with another vehicle. Drivers with TTPFs were characterized by more serious injuries than drivers without TTPFs, with these differences particularly noticeable in relation to chest injuries (Table 5).

### Analysis of the fall-from-heights group

*TTPFs were found in 41.7% (n = 14) of victims of falls from heights*. In eight out of those 14 cases and in 16 out of the 20 cases of falls from heights without TTPFs, the circumstances were known well enough to determine the approximate height from which the fall had occurred. *The lowest height a fall from which produced TTPFs was 9 m*, and the highest 30 m. The majority of persons with TTPFs had fallen from a height of more than 10 m. All fall-from-height TTPF victims had clear evidence of back injury. Among the victims of falls from heights without TTPFs, there were only two cases of falls from a height greater than 12 m, with one victim showing no signs of back injury and the other showing back injuries limited only to the subcutaneous tissue and muscles (without fractures of the scapula or transverse processes). The remaining falls in the non-TTPF group had occurred from a height of 12 m or less, most often from a height not higher than 10 m. Likewise, in the group of persons who died from falls from heights, the level of injury severity was higher in the TTPF than in the non-TTPF subgroup (Table 5), although the difference between these subgroups was not as significant as in the cases of pedestrian or driver deaths. Only one person (7.15%) from the fall-from-heights-with-TTPFs subgroup died in a hospital, whereas seven people (35%) from the corresponding non-TTPF subgroup did.

## Discussion

### Available literature on TTPFs

The scarcity of medicolegal publications on TPFs in general and TTPFs in particular indicates that a vast majority of forensic pathologists still perform autopsies in the traditional way and either fail to detect this type of injury or consider it noteworthy. A similar phenomenon had been commonplace in clinical practice before the growth in popularity of computed tomography examinations in traumatic cases [11], as TPFs were then thought to be rare [7] and to have no clinical significance [12]. Now due to an increased frequency in detecting TPFs, the interest in this type of injury has been increasing rapidly [13, 14]. Unfortunately, the majority of clinical publications are dedicated to TPFs of the lumbar vertebrae [10, 15, 16]. This is due to the fact that fractures of thoracic transverse processes are very rare in hospitalized patients [9, 13], which is consistent with our findings, which showed that the majority of people with TTPFs are found dead at the scene.

### TTPFs and internal injuries

Despite the fact that clinical publications focus more on TPFs in different parts of the vertebral column than our paper does, the essential conclusions are the same. Those include, first of all, the observation that transverse process fractures are accompanied by severe internal injuries [7, 8, 12, 15, 17–19]. In our study, TTPFs were most clearly associated with injuries to the heart and spleen. There were also a high proportion of TTPF cases with thoracic aortic injuries, but a statistical analysis showed a stronger association between aortic injuries and other types of thoracic spine fractures than TTPFs alone.

### Indirect mechanism leading to TTPFs

The results of our research indicate that several mechanisms may lead to TTPFs. *The most important one seems to be an indirect mechanism* where blunt trauma causes excessive thoracic flexion in the sagittal plane (the effect of which is comparable with that of a crushing injury). This is supported by our finding that in each case of trauma that resulted in TTPFs, rib fractures followed. Most often these were bilateral rib fractures. In addition, lung injuries were found in almost all cases; heart injuries were also present in a significant proportion of cases. Authors describing transverse process fractures in the lumbar segment of the spine also cite blunt trauma as one of the mechanisms that lead to these fractures (though in this case, it is blunt abdominal trauma leading to an increase in intra-abdominal pressure) [15]. Due to the differences both in the structure and in anatomical relations of the transverse processes in the thoracic and lumbar segments of the spine, the direct mechanism of TPFs as a result of trauma probably differs between these spinal segments. In the case of lumbar transverse processes, a rapid increase in intra-abdominal pressure causes tension of the muscles and tendons attached to these processes, which results in their avulsion [13, 15, 20, 21]. In at least some cases of TTPFs, it is reasonable to conclude that ribs play an important role as their posterior segments are connected to the transverse processes of the thoracic vertebrae and bend under the pressure of blunt trauma to the chest. This mechanism undoubtedly produces TTPFs defined in our paper as those “accompanying rib fractures.” Naturally, in order for TTPFs to occur, the intensity of blunt trauma (or crushing force) must be considerable, which also causes serious injuries to the internal organs of the chest. Apart from the ribs, the muscles attached to transverse processes also play a role in producing TTPFs [22, 23]. These muscles belong to the group of deep extensors of the thoracic spine. The function of these muscles is not only to straighten and stabilize the thoracic spine but also to support lateral spinal flexion [24]. The role of muscle tension in producing TTPFs is one of the theories that explain the observable difference in the incidence of this type of injury between drivers and passengers. Drivers tense more groups of muscles of the back and upper limbs than passengers do, especially when they see imminent obstacles. If this theory were to be proven, it would be of great practical significance in the cases where there is more than one victim of a traffic accident and there are doubts about who was driving the vehicle at the time of the accident. Only one publication indicates the potential use of detected TPFs of the upper thoracic and lower cervical spine in traffic accident reconstruction; however, the publication refers to the issue of determining whether or not the seatbelts had been fastened [25]. In our study, we were unable to check if the presence of TTPFs can be of use in differentiating the seatbelt-wearing from non-seatbelt-wearing victims because we did not have any data on seatbelt status. Apparently, tensing of the extensor muscles can also cause TTPFs in pedestrians, as this type of injury only occurred in those who had been hit antero-posteriorly, which is important when reconstructing the position of the victim’s body at the moment of collision. We were unable to confirm a relationship between the presence of TTPFs and the victim having been run over, which was described in one paper [26]. None of the pedestrians in our TTPF group had been literally run over by a car. This difference may be due to the fact that the mean age of the individuals evaluated in that study was considerably higher than in our study; moreover, that study evaluated not only pedestrians but also cyclists and motor scooter drivers.

### Direct mechanism leading to TTPFs

Another mechanism leading to TTPFs are direct injuries to the region of the processes themselves. This is indicated by the fact that signs of back injury were found in almost all TTPF cases, while in victims of falls from a considerable height (over 12 m), no TTPFs were found in the cases with no or poorly distinguishable signs of direct back injury. The possibility of such a mechanism leading to TPFs in the lumbar spine has been suggested by clinicians. Some authors also proved that transverse process fractures are produced by a weaker force when it acts directly on the person’s back than when such fractures are produced by any other mechanism [13]. This observation is consistent with the results of a cadaver study that showed that breaking lumbar transverse processes requires a two times stronger force to be applied to the sides of the body than to the back [27]. However, we were unable to confirm this phenomenon with respect to TTPFs because our study involved only high-energy trauma cases. In our study, there was one case of an isolated fracture (a single transverse process of vertebra T12) which was the result of an impact with an excavator bucket. In light of publications which indicate that the structure of the last two thoracic vertebrae is more similar to the lumbar vertebrae than to the other distal thoracic vertebrae [28], this isolated fracture can be assumed to be a result of direct trauma to this area, as it was described in lumbar TPFs.

## Conclusions

The presence of TTPFs indicates an application of a very powerful force, which (in most cases) leads to injuries resulting in death at the scene of the event. The most common mechanism producing TTPFs appears to be trauma crushing the chest in the antero-posterior dimension, with direct trauma to the back and tensing of the muscles attached to the transverse processes also possibly playing a role. Detection of TTPFs provides additional information about the mechanism of trauma, especially in pedestrians, drivers, passengers, and victims of falls from heights.
